# The lasting effects of resistance and endurance exercise interventions on breast cancer patient mental wellbeing and physical fitness

**DOI:** 10.1038/s41598-022-07446-3

**Published:** 2022-03-03

**Authors:** Jonathon Mok, Marie-Juliet Brown, Elizabeth C. Akam, Mhairi A. Morris

**Affiliations:** School of Sport, Exercise and Health Sciences, Loughborough University, Towers Way, Loughborough, LE11 3TU UK

**Keywords:** Breast cancer, Breast cancer

## Abstract

Breast cancer is a persisting global burden for health services with cases and deaths projected to rise in future years. Surgery complemented by adjuvant therapy is commonly used to treat breast cancer, however comes with detrimental side effects to physical fitness and mental wellbeing. The aim of this systematic review and meta-analysis is to determine whether resistance and endurance interventions performed during adjuvant treatment can lastingly ameliorate these side effects. A systematic literature search was performed in various electronic databases. Papers were assessed for bias and grouped based on intervention design. RStudio was used to perform the meta-analyses for each group using the ‘meta’ package. Publication bias and power analyses were also conducted. These methods conform to PRISMA guidelines. Combined resistance and endurance interventions elicited significant long-lasting improvements in global fatigue and were beneficial to the remaining side effects. Individually, resistance and endurance interventions non-significantly improved these side effects. Resistance interventions elicited higher benefits overall. Exercise interventions have lasting clinical benefits in ameliorating adjuvant therapy side effects, which negatively impact physical fitness and mental wellbeing. These interventions are of clinical value to enhance adherence rates and avoid comorbidities such as sarcopenia, thus improving disease prognosis.

## Introduction

In 2020, there were approximately 19.3 million new cancer cases globally, of which, female breast cancer was the highest contributor at 11.7% of the total. As a result of these new cases, there were 10 million deaths attributed to cancer: female breast cancer constituted 6.9% of these deaths (684,996 deaths)^1^. The number of new breast cancer cases and mortality rates are only projected to rise in future years, thus female breast cancer represents a significant burden on female health and health services.

Currently, multiple treatment options exist to treat breast cancer. These primarily involve surgery to remove the tumour, usually a mastectomy or breast conserving surgery, which are followed up by adjuvant therapy such as chemotherapy, radiotherapy, hormone therapy or forms of targeted therapy^2^. This ensures the tumour is removed and the risk of relapse is reduced due to a decreased risk of metastasis that results from adjuvant therapy^3^. In 2019, breast conserving surgery followed up with adjuvant radiotherapy was the most common form of treatment for early breast cancer in stages I and II in American female breast cancer patients (49%)^4^. For more severe breast cancers in stages III and IV, chemotherapy and hormonal therapy were the most common form of treatments in American female patients—56% and 71% of all cases were treated with these, respectively^4^.

While adjuvant therapy has shown much success in recent years by extending overall survival and disease-free survival in breast cancer patients^5^, adjuvant treatment such as taxane-based chemotherapy also causes various unwanted life-changing side effects. Common side effects include disturbances to mental wellbeing manifested in depression and fatigue, leading to an overall decreased Quality of Life (QOL)^6^. Other well-documented side effects include declines in physical fitness, manifested in reduced muscular strength and endurance following treatment^7^. These may decrease physical capacity and therefore daily physical functioning, which may also contribute to decreased adherence to treatment, ultimately decreasing the efficacy of adjuvant treatment. These side effects are therefore important to manage and enhance adherence rates boosting the efficacy of treatment options and therefore disease prognosis.

Generally, exercise is well characterised to reduce the risk of developing breast cancer and to reduce the mortality rates linked to breast cancers. McTiernan et al*.*^8^ show that the risk of developing breast cancer is reduced by up to 18% when exercise is performed regularly. Alongside this, Palesh et al*.*^9^ demonstrated that an hour a day of moderate physical activity decreases the mortality of advanced breast cancers by 23%. Specifically, resistance and endurance exercise designs are typically used in the array of studies investigating the effects of exercise on breast cancer survival and risk. Resistance exercise is defined as using resistance in the form of weights or resistance bands to elicit muscular hypertrophy^10^ whereas endurance exercise is the continuous activation of skeletal muscle groups over a prolonged period of time to improve aerobic capacity^11^.

While many reviews have characterised the beneficial effects of exercise on breast cancer survival and mortality, no reviews to date have quantified the effects of resistance and endurance interventions to ameliorate the detrimental side effects impacting physical fitness and mental wellbeing that come with adjuvant therapy in order to avoid further pathology and improve daily functioning which may boost the efficacy of these treatments. In addition, whether the beneficial effects of exercise to ameliorate these side effects are lasting is yet to be elucidated.

Therefore, the aims of this meta-analysis and systematic review are:

1. To quantify the lasting effects of combined resistance and endurance interventions on physical fitness and mental wellbeing in female breast cancer patients (≥ 18 years old) undergoing adjuvant therapy by measuring the following factors: cardiorespiratory fitness, depression, fatigue, muscular endurance, muscular strength, QOL and social functioning.
2. To quantify the lasting effects of interventions consisting of only resistance or only endurance exercise on these factors and to elucidate which type of exercise is more effective (by comparison) in improving mental wellbeing and physical fitness in patients undergoing adjuvant therapy.

## Materials and methods

### Search method

This systematic review and meta-analysis conforms to the Preferred Reporting Items for Systematic Reviews and Meta-Analyses (PRISMA) guidelines^12^. To obtain papers for this meta-analysis, a comprehensive systematic literature search was conducted in the following electronic databases: PubMed, BioMed Central (BMC), Scopus, Web of Science Core collection, Cochrane Library and Ovid with the last search being conducted in December 2020. Search terms to obtain these papers used the Boolean operator “AND” to narrow the results returned and terms started off broadly such as “exercise” AND “cancer” to identify the knowledge gap in the field of exercise oncology. These terms progressively became more specific to pinpoint required papers to answer the knowledge gap. Specific search terms included “endurance” AND “resistance exercise” “on breast cancer”. A full list of search terms used to conduct the literature search are listed in Table 4 in the Supplementary Materials. The inclusion criteria to select these papers is as follows: study was a published randomised controlled trial; a published clinical trial with a complete dataset; used human participants; contained endurance/aerobic or resistance exercise interventions lasting a minimum of 20 min per session; investigated at least one of the outcome measures required; was written in English; was published from 2010 to 2020; is exclusive to breast cancer; is a 4 + star paper (OVID). The OVID star ranking relates to how relevant the papers are according to the search terms inputted: 4 + stars were used to filter out irrelevant papers that would have no value to these meta-analyses.

### Outcome measures

Outcome measures obtained from each study that met the inclusion criteria were cardiorespiratory fitness, depression, global fatigue, muscular endurance, muscular strength, QOL and social functioning. Cancer related fatigue was used as a substitute where global fatigue was not measured. Cardiorespiratory fitness, muscular endurance and muscular strength constitute the umbrella term “physical fitness”, and depression, global fatigue, QOL and social functioning constitute the umbrella term “mental wellbeing”. These were all continuous outcomes. Further information about each of these characteristics is given in Table 1.

**Table 1:** Information to further define the outcome measures used in these meta-analyses including which studies they were used in, how they were collected by each study and what type of data they are.

Outcome measure	Study	Method used to collect data	Type of data
Cardiorespiratory fitness	Cornette et al.^13^	Cardiopulmonary Exercise Test (CPET)	Mean
	Dong et al.^14^	Modified Bruce treadmill protocol	Mean
	Cornette et al.^15^	CPET	Mean
	Travier et al.^16^	CPET	Mean
	Waart et al.^17^	Steep ramp test	Mean
	Casla et al.^18^	Modified Bruce treadmill protocol	Mean
	Al-Majid et al.^19^	Modified Bruce treadmill protocol	Mean
	Bolam et al.^20^	Astrand-Rhyming submaximal cycling test	Mean
	An et al.^21^	Maximal incremental exercise treadmill protocol	Mean
Depression	Cornette et al.^13^	Hospital Anxiety and Depression Scale (HADS)	Mean
	Travier et al.^16^	HADS	Mean
	Schmidt et al.^22^	Centre for Epidemiological studies depression scale (CES-D)	Mean
	Steindorf et al.^23^	CES-D	Mean
	Courneya et al.^24^	CES-D	Mean
Global fatigue	Cornette et al.^13^	Multidimensional Fatigue Inventory (MFI-20)	Mean
	Travier et al.^16^	MFI-20 and Fatigue Quality List (FQL)	Mean
	Waart et al.^17^	MFI-20 and FQL	Mean
	Husebø et al.^25^	Schwartz Cancer Fatigue Scale (SCFS-6)	Mean
	Schmidt et al.^26^	European Organisation for Research and Treatment of Cancer QLQ-C30 BR23 (EORTC QLQ-C30 BR23)	Mean
	Schmidt et al.^22^	EORTC QLQ-C30 BR23	Mean
	Cešeiko et al.^27^	EORTC QLQ-C30 BR23	Mean
	Steindorf et al.^23^	Fatigue Assessment Questionnaire (FAQ)	Mean
	Al-Majid et al.^19^	Piper Fatigue Scale (PFS)	Mean
	Bolam et al.^20^	PFS	Mean
	Schmidt et al.^28^	MFI-20	Mean
Muscular endurance	Cešeiko et al.^29^	Submaximal walking time to exhaustion	Mean
	Schmidt et al.^28^	Endurance stress test W/KG/Bodyweight	Mean
	An et al.^21^	Repetitions of 50% or 70% 1 rep-max in chest and leg press	Mean
Muscular strength	Cornette et al.^13^	3 repetition max knee flexion	Mean
	Dong et al.^14^	Chair stand test	Mean
	Travier et al.^16^	Knee extension 1 Rep Max (1RM)	Mean
	Waart et al.^17^	Knee extension (Nm)	Mean
	Casla et al.^18^	Leg extension 1RM	Mean
	Cešeiko et al.^29^	Leg press 1RM	Mean
	Wiskemann et al.^30^	Knee extension	Mean
	Bolam et al.^20^	Isometric thigh pull	Mean
	Schmidt et al.^28^	Leg press (Nm)	Mean
	An et al.^21^	Leg press 1RM	Mean
Quality of life	Cornette et al.^13^	EORTC QLQ-C30	Mean
	Travier et al.^16^	EORTC QLQ-C30	Mean
	Casla et al.^18^	SF-36	Mean
	Schmidt et al.^26^	EORTC QLQ-C30 BR23	Mean
	Schmidt et al.^22^	EORTC QLQ-C30	Mean
	Cešeiko et al.^27^	EORTC QLQ-C30 BR23	Mean
	Steindorf et al.^23^	EORTC QLQ-C30 BR23	Mean
	Al-Majid et al.^19^	Functional Assessment of Cancer Therapy Breast (FACT-B)	Mean
	Bolam et al.^20^	EORTC QLQ-C30	Mean
	Schmidt et al.^28^	EORTC QLQ-C30 BR23	Mean
Social functioning	Dong et al.^14^	SF-36	Mean
	Cešeiko et al.^27^	EORTC QLQ-C30	Mean
	Bolam et al.^20^	EORTC QLQ-C30	Mean
	Schmidt et al.^28^	EORTC QLQ-C30 BR23	Mean

### Data extraction and risk of bias

Data (means, standard deviations and numbers of participants) concerning the above outcome measures were extracted from baseline and from the last available time points in each study that reached the inclusion criteria from both the exercise intervention and control conditions (Table 2). If the data was not immediately available, the corresponding authors were contacted directly via email requesting the relevant data. If the authors were unable to reply, their papers were excluded from the meta-analyses. Each paper reaching the inclusion criteria was assessed for risk of bias using the National Toxicology Program’s Office of Health Assessment and Translation (OHAT) Risk of Bias rating tool^31^. The questions used for assessment are as follows: (1) Was administered dose or exposure level adequately randomised? (2) Was allocation to study groups adequately concealed? (3) Did selection of study participants result in appropriate comparison groups? (4) Did the study design or analysis account for important confounding and modifying variables? (5) Were the research personnel and human subjects blinded to the study group during the study? (6) Were outcome data complete without attrition or exclusion from analysis? (7) Can we be confident in the exposure characterization? (8) Can we be confident in the outcome assessment? (9) Were all measured outcomes reported? (10) Were there no other potential threats to internal validity? Risk of bias analysis was also carried out by another researcher using the OHAT tool to avoid bias which may arise from singular opinion. To gain an overall rating for each study, a numerical system was deployed which takes into account each question equally. In this, the rating “Definitely low” risk equated to + 4 points, “Probably low” risk equated to + 2, “Definitely High” risk equated to − 4 and “Probably low” risk equated to − 2 points. An average of these scores was then taken for each study across the 10 domains. If the average score was between + 2 to + 4 the rating was “Definitely low” risk, + 0.1 to + 1.9 was “Probably low” risk, − 2 to − 4 was “Definitely High” risk and 0 to − 1.9 was “Probably high” risk. The rating “NR” was excluded from this average calculation. Disparities in the rating of the studies were resolved by discussion and a consensus was reached. The risk of bias analysis evaluates the papers on their ability to minimise potential sources of bias that may negatively impact results and therefore scientific validity. In combination, the star ranking, and risk of bias analysis, ensures the most appropriate studies were used for this meta-analysis.

**Table 2:** Study characteristics for studies that were used in these meta-analyses including Participant numbers at baseline, adjuvant therapy type, type of exercise intervention used, intervention details, intervention duration, end follow up time point used, ages of participants in each study and which outcome measures the studies contributed to.

Study	Participant numbers at baseline	Adjuvant therapy type	Type of exercise intervention	Intervention details	Intervention Duration (Weeks)	End follow up timepoint used in analysis (Weeks)	Ages of participants (Years)	Outcomes reported in this meta-analysis
Cornette et al.^13^	Intervention (N = 20) Control (N = 22)	Chemotherapy (Neoadjuvant or adjuvant) followed by radiotherapy	Resistance and endurance	Randomized 27-week home-based exercise program combining strength and endurance training throughout adjuvant chemotherapy lasting up to 40 min per session, 3 times per week	54	54	18–75	Cardiorespiratory fitness (VO_2_peak), Depression, Global fatigue, Lower body muscular strength (1 RM leg) and Quality of Life (QOL)
Dong et al.^14^	Intervention (N = 30) Control (N = 30)	Chemotherapy/postoperative radiotherapy	Resistance and endurance	Randomized 12-week internet-based exercise intervention consisting of resistance and endurance training lasting 30 min per session, 3 times per week. Followed by 40 weeks of unsupervised exercise intervention	12	52	43–59	Cardiorespiratory fitness (VO_2_peak), Lower body muscular strength (chair stand test), Social Functioning
Cornette et al.^15^	Intervention (N = 22) Control (N = 22)	Adjuvant or neoadjuvant chemotherapy and radiotherapy	Resistance and endurance	Randomized 27-week home-based exercise program combining strength and endurance training throughout adjuvant chemotherapy and radiotherapy	54	54	40–64	Cardiorespiratory fitness (VO_2_peak)
Travier et al.^16^	Intervention (N = 102) Control (N = 102)	Chemotherapy	Resistance and endurance	Randomized 18-week exercise program consisting of 2 endurance and strength exercise sessions weekly lasting 60 min per session. Each session was supervised by a physiotherapist	18	36	25–75	Cardiorespiratory fitness (VO_2_peak), Depression, Global Fatigue, Lower body muscular strength (left knee flexor peak torque at 60 degrees/s (nm)), QOL
Waart et al.^17^	Intervention (N = 76) Control (N = 77)	Chemotherapy	Resistance and endurance	Randomized resistance and endurance exercise program (OnTrack) twice weekly lasting 50 min per session. Supervised by physical therapists	Until 3 weeks after the final chemotherapy cycle	26 weeks after final cycle of chemotherapy	41–59	Cardiorespiratory fitness (Endurance time, minutes), Global fatigue and Lower body muscular strength (Knee extension Nm)
Casla et al.^18^	Intervention (N = 47) Control (N = 47)	Chemotherapy and radiotherapy	Resistance and endurance	Randomized resistance and endurance exercise program based on the ACSM guidelines twice weekly. Complemented with an educational program about nutrition and exercise guidelines	12	26 weeks after program completion	18 +	Cardiorespiratory fitness (VO_2_max), Lower body muscular strength (Maximal strength legs/Weight) and QOL (SF36 physical)
Husebø et al.^25^	Intervention (N = 33) Control (N = 34)	Chemotherapy	Resistance and endurance	Randomized home-based strength and aerobic training lasting at least 30 min per session, 3 times per week	24	50	18–70	Cancer related fatigue
Schmidt et al.^26^	Intervention (N = 15) Control (N = 18)	Chemotherapy and radiotherapy	Resistance and endurance	Randomized strength endurance training consisting of 20 repetitions at 50% 1RM lasting 1 h per session, once weekly	26	26	18–70	Global Fatigue and QOL
Schmidt et al.^22^	Intervention (N = 49) Control (N = 49)	Chemotherapy	Resistance	Randomized progressive machine-based resistance training lasting 60 min per session twice weekly. Supervised by experienced therapists	12	13	18 +	Depression, Global fatigue and QOL
Cešeiko et al.^27^	Intervention (N = 27) Control (N = 28)	Chemotherapy or radiotherapy or hormone therapy	Resistance	Randomized maximal strength training twice weekly	12	12	18–63	Global Fatigue, QOL (Global health status) and social functioning
Steindorf et al.^23^	Intervention (N = 80) Control (N = 80)	Radiotherapy	Resistance	Randomized progressive machine-based resistance exercise 60 min per session twice weekly	12	13	18 +	Global Fatigue, QOL and depression
Cešeiko et al.^29^	Intervention (N = 27) Control (N = 28)	Radiotherapy or chemotherapy	Resistance	Randomized resistance training through 90% of 1RM twice weekly	12	12	18–63	Lower body muscular endurance (Time to Exhaustion) and Lower body muscular strength (Leg press 1RM)
Wiskemann et al.^30^	Intervention (N = 80) Control (N = 80)	Radiotherapy	Resistance	Randomized machine-based progressive resistance exercise 3 sets of a 12-repetition maximum	12	13	18 +	Lower body muscular strength (Knee flexion (60°))
Al-Majid et al.^19^	Intervention (N = 7) Control (N = 7)	Chemotherapy	Endurance	Randomized progressive endurance program on treadmill lasting at least 20 min, 2–3 times weekly	12	16	21 +	Cardiorespiratory fitness (VO_2_max), Cancer related fatigue, QOL (FACT-B total)
Bolam et al.^20^	Intervention (N = 74) Control (N = 60)	Chemotherapy	Resistance vs Usual care and Endurance vs Usual care	Randomized resistance training using both machines and free weights. Endurance training with moderate intensity continuous aerobic exercise. Both training groups lasted 60 min per session, twice weekly	16	104	18–70	Cardiorespiratory fitness (VO_2_peak), Global Fatigue, Lower body muscular strength (Isometric mid-thigh pull), QOL and social functioning
Schmidt et al.^28^	Intervention (N = 21) Control (N = 26)	Chemotherapy	Resistance vs Usual care and Endurance vs Usual care	Randomized resistance training using 20 repetitions of 50% 1RM on multiple machines. Endurance training using an indoor bike. Both sessions lasted 60 min taking place twice weekly	12	24	41–66	Global Fatigue, Lower body muscular endurance (W/KG/BW), Lower body muscular strength (Leg press), QOL and social functioning
Courneya et al.^24^	Intervention (N = 104) Control (N = 96)	Chemotherapy	Endurance and Resistance vs just endurance	Randomized combined dose of resistance and endurance exercise lasting 50- 60 min vs just 25–30 min of endurance exercise. Both done 3 times weekly	Doesn’t say	3–4 weeks after chemotherapy finished	18 +	Depression
An et al.^21^	Intervention (N = 104) Control (N = 96)	Chemotherapy	Endurance and Resistance vs just endurance	Randomized combined dose of resistance and endurance exercise lasting 50- 60 min vs just 25–30 min of endurance exercise. Both done 3 times weekly	18	104	≥ 18	Cardiorespiratory fitness (VO_2_peak), Fatigue, Lower body muscular endurance (repetitions), and Lower body muscular strength

### Data handling and statistical analysis

**Figure 1:**
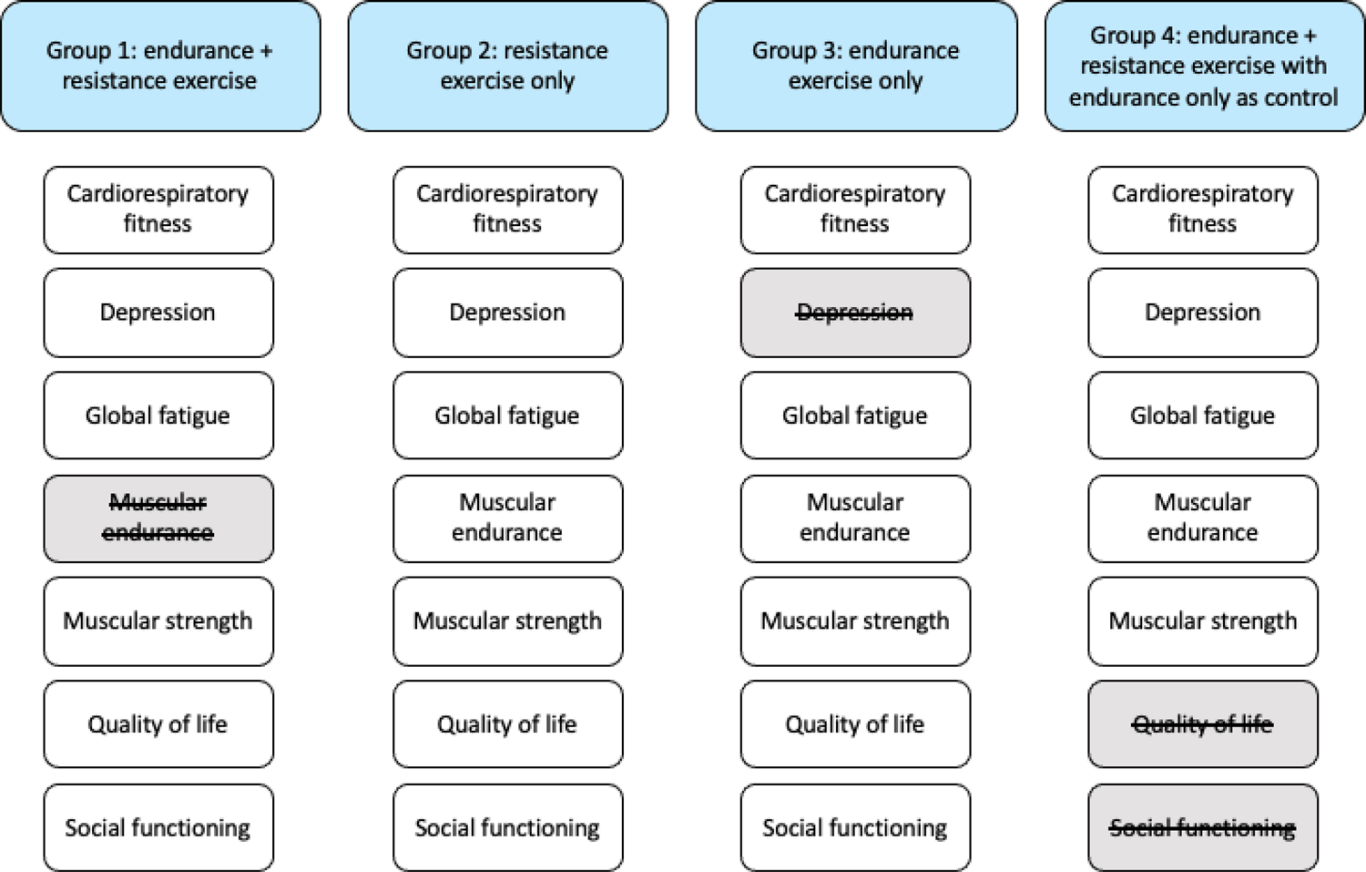
Graphical representation of how papers were sorted in data handling to use in their respective meta-analyses. Grey shading and score-through represent groupings for which there were not enough studies to analyse certain outcome measures.

Once all the necessary data was extracted, papers were sorted into 4 groups by intervention design to answer the aims of this study. The exercise interventions were defined using experimental details provided by the papers used (Table 2). The first group consisted of papers with interventions that used both resistance and endurance exercise. The second group consisted of papers with an exercise intervention consisting of solely resistance exercise while the third group consisted of papers with endurance interventions only. A fourth (comparative) group was established which consisted of papers that compared resistance exercise to endurance exercise by using interventions consisting of both resistance and endurance as the intervention condition and interventions with just endurance as the control. Within these groups, the papers were grouped again by which of the outcome measures they investigated. Using these categories, a meta-analysis was carried out for each factor in each of the 4 groups. This allowed the investigation of the effects of having both resistance and endurance exercise on the outcome measures, the effects of having just resistance or endurance exercise and the effects of adding resistance to endurance exercise on the outcome measures (to further quantify which design was more effective) respectively. This process is graphically presented in Fig. 1. Interventions that were solely resistance, were characterised by being high intensity over a short duration with the sole aim of improving muscular strength, whereas solely endurance interventions were low intensity over a longer duration with the sole aim of improving VOmax. Combined interventions consisted of a mix of these characteristics^32^.

To conduct the meta-analyses, RStudio was used^33^. Within RStudio, the ‘meta’ package was loaded enabling the ‘metacont’ function to be used to calculate effect sizes and 95% confidence intervals of each study. The summary measure used was Standardised Mean Difference (SMD) with Hedges’ g correction with the Q-profile method being used to calculate confidence intervals. Prediction intervals were also calculated for each factor where available, with both fixed and random models being generated for these analyses, to ensure transparency in statistical analysis. The most appropriate model from these two was then chosen for each analysis. This was determined by the percentage of variability in effect sizes across studies (I^2^):—if heterogeneity was present between the studies being analysed (> 0%), the random effects model was reported because having heterogeneity between studies indicates there is not uniform contribution to the overall effect size. Thus, reporting the random effects model where appropriate, captures this assumption allowing the most accurate outcome to be utilised. When using the random effects model the Hartung-Knapp adjustment was applied to minimise Type 1 error rates^34^. The Inverse Variance method was used in each meta-analysis to calculate the weight/contribution of each study to the overall effect size displayed. To quantify between-study variance (Tau^2^), Restricted Maximum-Likelihood (REML) was used due to being low in bias and yielding low Mean Squared Errors (MSE) of Tau^2^ for the number of studies and sample sizes used in these meta-analyses^35^. In the event that REML could not converge on a Tau^2^ estimate, the Sidik-Jonkman-type estimator (SJ) was used as an alternative due to having low bias estimates of Tau^2^. To summarise this data, forest plots were created for each variable using the ‘forest.meta’ function within the ‘meta’ package.

To investigate publication bias, a funnel plot was constructed using the ‘meta’ package and ‘funnel’ function, encompassing all of the studies included in the meta-analyses. To statistically quantify this, the Egger’s test of intercept was calculated using the ‘dmetar’ package enabling the use of the function ‘eggers.test’. Assessing for publication bias ensures the true effect sizes calculated are representative and not inflated due to studies finding small effect sizes not being published/included.

For each meta-analysis conducted, power analysis was carried out to quantify whether there was sufficient power to detect a statistically significant effect size where one exists. This was performed using the ‘power.analysis’ function as part of the ‘dmetar’ package^36^. Where random-effects models were reported, heterogeneity levels (I^2^) for usage in the power calculation were defined using the following categories: 25% = Low, 50% = Moderate and 75% = High^37^.

## Results

### Study selection

**Figure 2:**
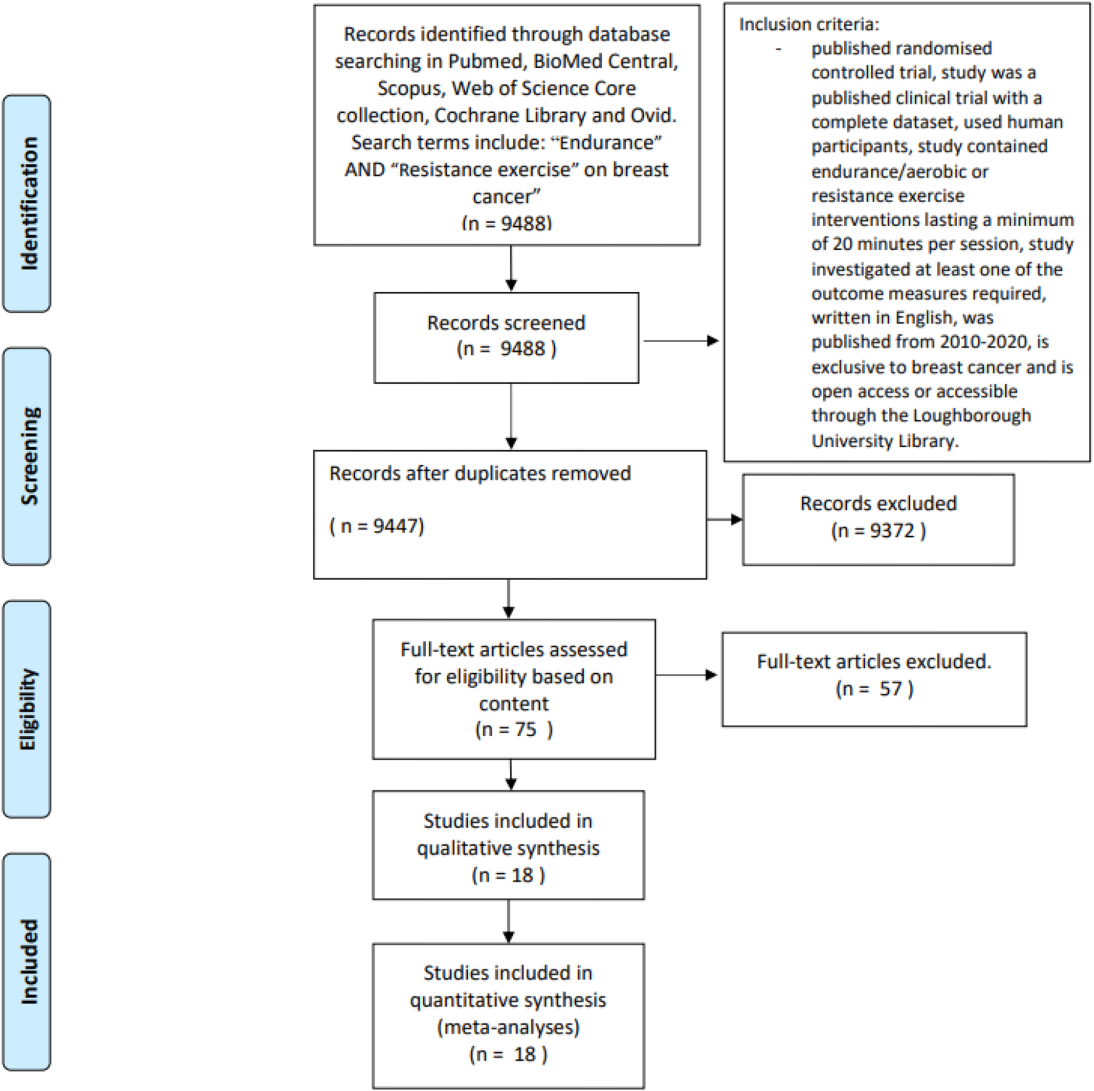
PRISMA 2009 flow diagram detailing the step-wise process used for study selection for these meta-analyses.

Once the gap in research was identified, 9488 papers were first obtained using the search terms “endurance” AND “resistance exercise” “on breast cancer”. These papers were screened to check if they met the inclusion criteria stated previously and if the abstract, intervention design and outcome measures were relevant to these meta-analyses. Of these papers, 9372 were removed. 41 duplicate papers were also removed. This left 75 full-text papers which were assessed for eligibility based on content. This resulted in a further 57 papers being excluded, with one of these papers being unable to provide the data required, leaving 18 papers to be used in these meta-analyses. This process is shown in Fig. 2. For each variable assessed, the largest number of studies possible that could contribute to these meta-analyses were included, obtained using rigorous search terms. Conducting analysis where there was only one or two studies allows us to demonstrate the need for further research in these areas, while attempting to provide an insight into how exercise may impact physical fitness and mental wellbeing in breast cancer patients receiving adjuvant therapy. This may provide a valuable baseline for future studies to use, to further progress literature in this area.

### Study sorting

The 18 selected papers were sorted into their respective groups using the method described previously, to perform the meta-analyses required. This is shown in Table 5 in the Supplementary Materials.

### Risk of bias

**Figure 3:**
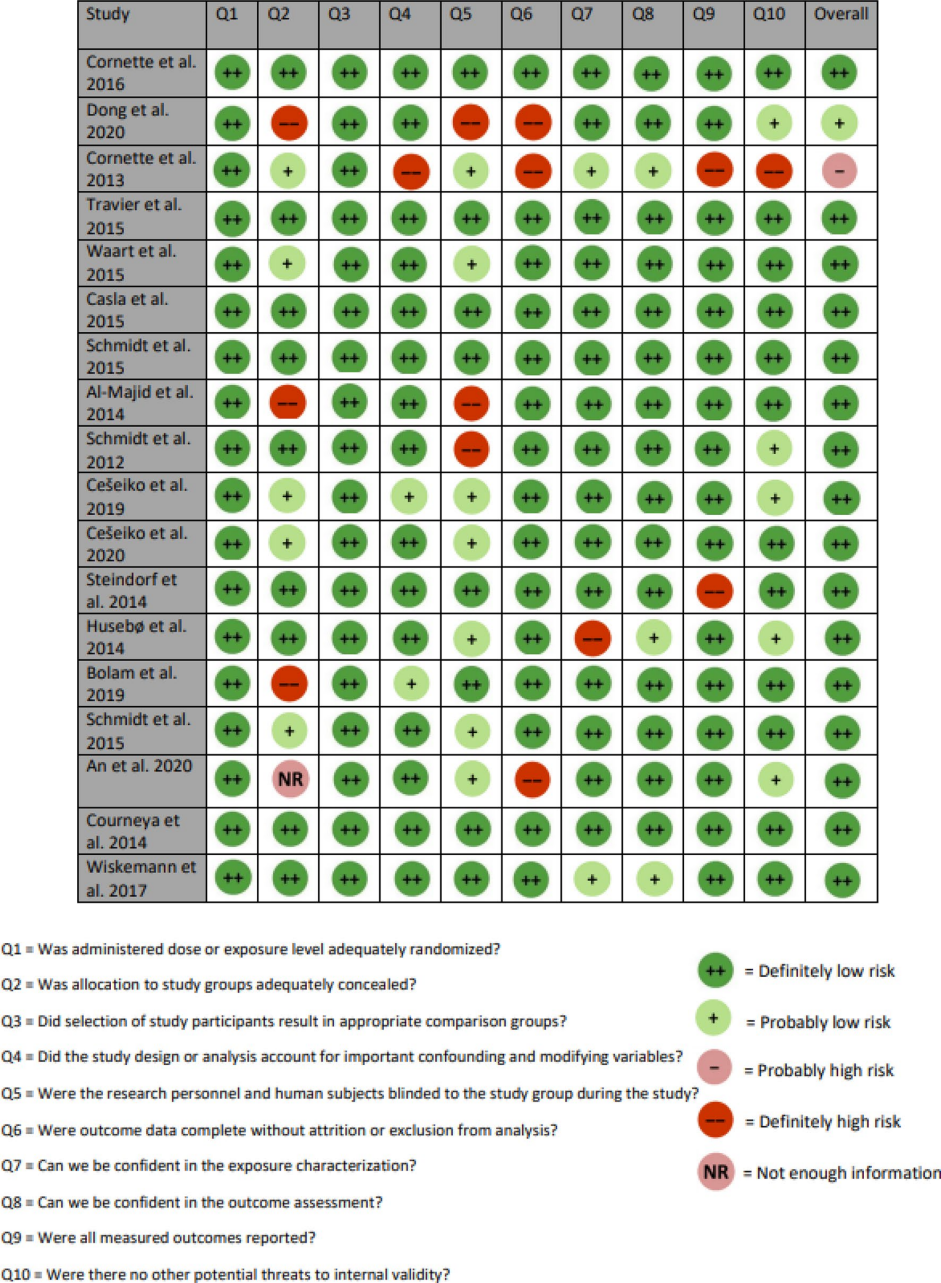
Risk of bias results for the 18 studies included in the meta-analyses using the OHAT rating tool.

Of the 18 papers selected, 16 were shown to be “definitely low” in risk when considering all 10 questions. One paper was shown to be “probably high” in risk while the other paper was “probably low” in risk (Fig. 3).

### Publication bias

**Figure 4:**
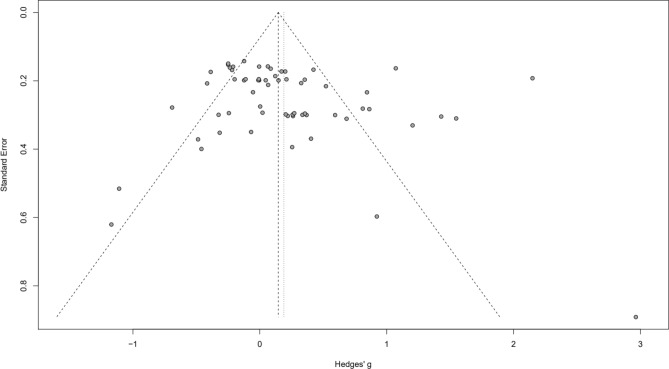
Funnel plot showing symmetry and therefore no publication bias in the papers used.

The studies used for each factor and group exhibited no publication bias. This was shown by funnel plot symmetry and was statistically confirmed by Egger’s test being non-significant (*P* = 0.176). This is shown in Fig. 4.

### Meta-analyses

#### Combined interventions comprising both resistance and endurance exercise: cardiorespiratory fitness

**Figure 5:**
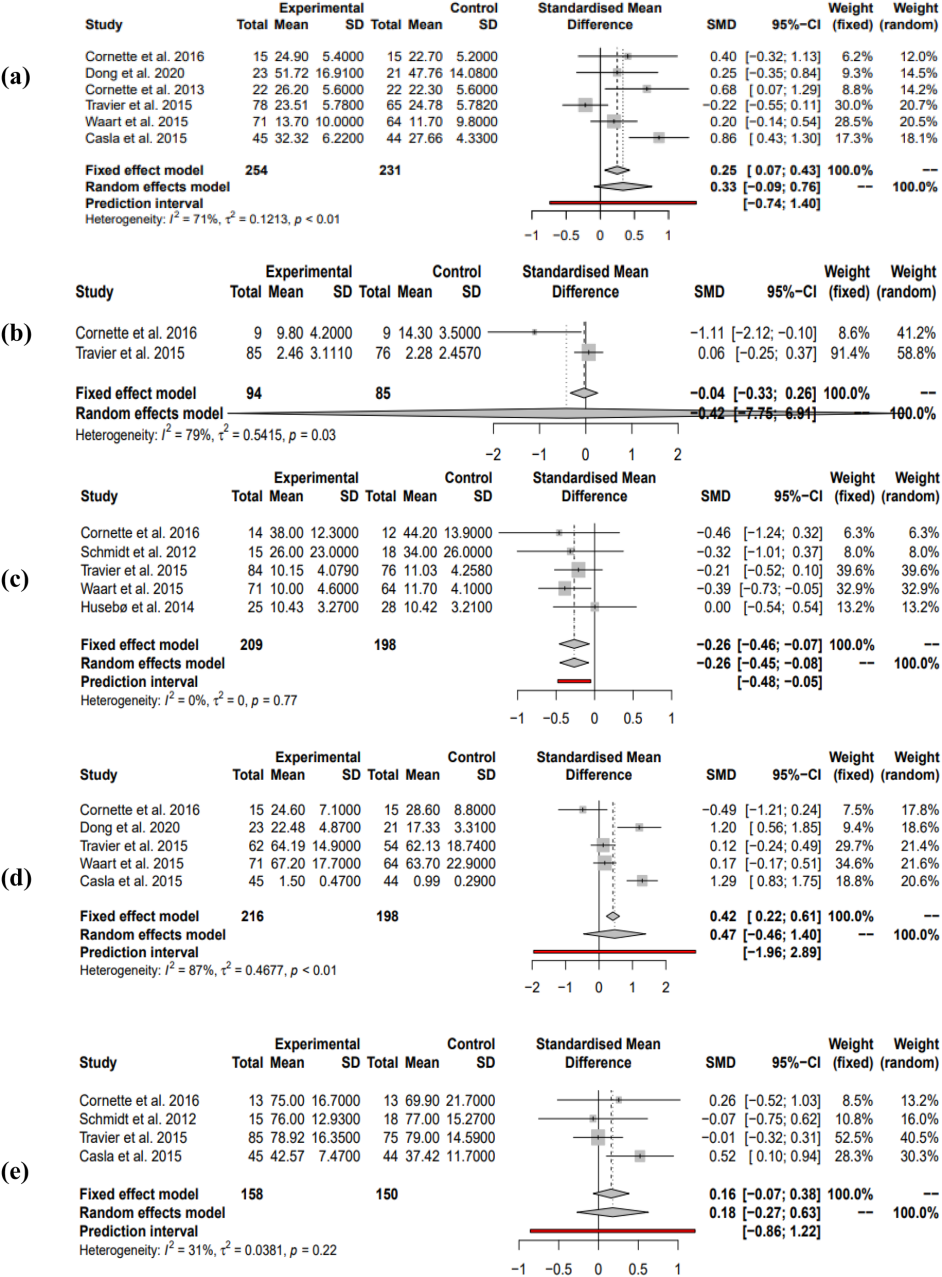
Random effects models showing the effects of combined resistance and endurance interventions on (**a**) cardiorespiratory fitness, (**b**) depression, (**d**) muscular strength, (**e**) QOL during adjuvant treatment. Fixed effects model showing the effects of combined resistance and endurance interventions on (**c**) global fatigue during adjuvant treatment. Positive effect sizes favour the exercise intervention in (**a,d,e**). Negative effect sizes favour the exercise intervention in (**b,c**).

Of the 18 studies selected^13–18^, six were used to investigate the effects of combined interventions consisting of both resistance and endurance exercise on cardiorespiratory fitness in women undergoing adjuvant therapy. Five out of six studies showed a positive effect size while one study showed a low negative effect size. Collectively, using the random effects model due to high heterogeneity, the overall effect size was non-significant low positive (SMD = 0.33, 95% CI = [− 0.09; 0.76], I^2^ = 71%, *P* = 0.09). Power analysis revealed this meta-analysis to have an optimal level of power of 80.23%. Prediction intervals suggest future studies will favour a positive effect size (Fig. 5a).

#### Combined interventions comprising both resistance and endurance exercise: depression

Two studies^13,16^ were used to quantify the effects of combined exercise interventions on depression. Out of the two studies, one showed a large negative effect size while the other showed a low positive. Reporting the random effects model, the overall effect size was found to be non-significant low negative (SMD = − 0.42, 95% CI = [− 7.75; 6.91], I^2^ = 79%, *P* = 0.60). Power analysis shows this meta-analysis to have low power to detect a statistically significant effect size where one exists at 50.35%. Due to only being able to use two studies, prediction intervals could not be created. This is shown in Fig. 5b.

#### Combined interventions comprising both resistance and endurance exercise: global fatigue

Five studies^13,16,17,25,26^ were used to investigate the effects of combined exercise interventions on global fatigue. All but one study showed a negative effect size, with the remaining one showing no effect. Collectively, a significant negative effect size was found when reporting the fixed effects model due to a lack of heterogeneity found by both REML and SJ (SMD = − 0.26, 95% CI = [− 0.46; − 0.07], I^2^ = 0%, *P* = 0.008). This was however accompanied by less-than-optimal statistical power (74.55%). Prediction intervals suggest this will be also found in future studies (Fig. 5c).

#### Combined interventions comprising both resistance and endurance exercise: muscular endurance

No studies could be found with the desired inclusion criteria that investigated the effects of combined interventions on muscular endurance.

#### Combined interventions comprising both resistance and endurance exercise: muscular strength

Four of the five studies^13,14,16–18^ used to investigate the effects of combined resistance and endurance interventions on muscular strength found positive effect sizes. Collectively, using the random effects model these studies showed a small non-significant positive effect size (SMD = 0.47, 95% CI = [− 0.46; 1.40], I^2^ = 87%, *P* = 0.235). Prediction intervals also favour a positive effect size in future studies. Optimal power was also achieved in this meta-analysis (91.86%). This is demonstrated in Fig. 5d.

#### Combined interventions comprising both resistance and endurance exercise: QOL

Overall^13,16,18,26^, a non-significant low positive effect size was observed with low heterogeneity using the random effects model but with low statistical power of 27.98% (SMD = 0.18, 95% CI = [− 0.27; 0.63], I^2^ = 31%, *P* = 0.295). Prediction intervals also support this indicating a positive effect size will likely to be found in future studies (Fig. 5e).

#### Combined interventions comprising both resistance and endurance exercise: social functioning

Only one study was available to quantify the effects of combined resistance and endurance interventions on social functioning. Dong et al*.*^14^ showed a non-significant positive effect size (SMD = 0.26, 95% CI = [− 0.33; 0.86], *P* = 0.39). Power analysis and prediction intervals could not be carried out.

#### Interventions comprising solely resistance exercise: cardiorespiratory fitness

Only one study could be found with the desired inclusion criteria that investigated the lasting effects of solely resistance interventions on cardiorespiratory fitness during adjuvant treatment. Bolam et al.^20^ showed a non-significant low positive effect size favouring the resistance intervention (SMD = 0.21, 95% CI = [− 0.17;0.59], *P* = 0.283). The direction of future studies however is unclear due to not being able to generate prediction intervals. In addition, power analysis could not be carried out.

#### Interventions comprising solely resistance exercise: depression

**Figure 6:**
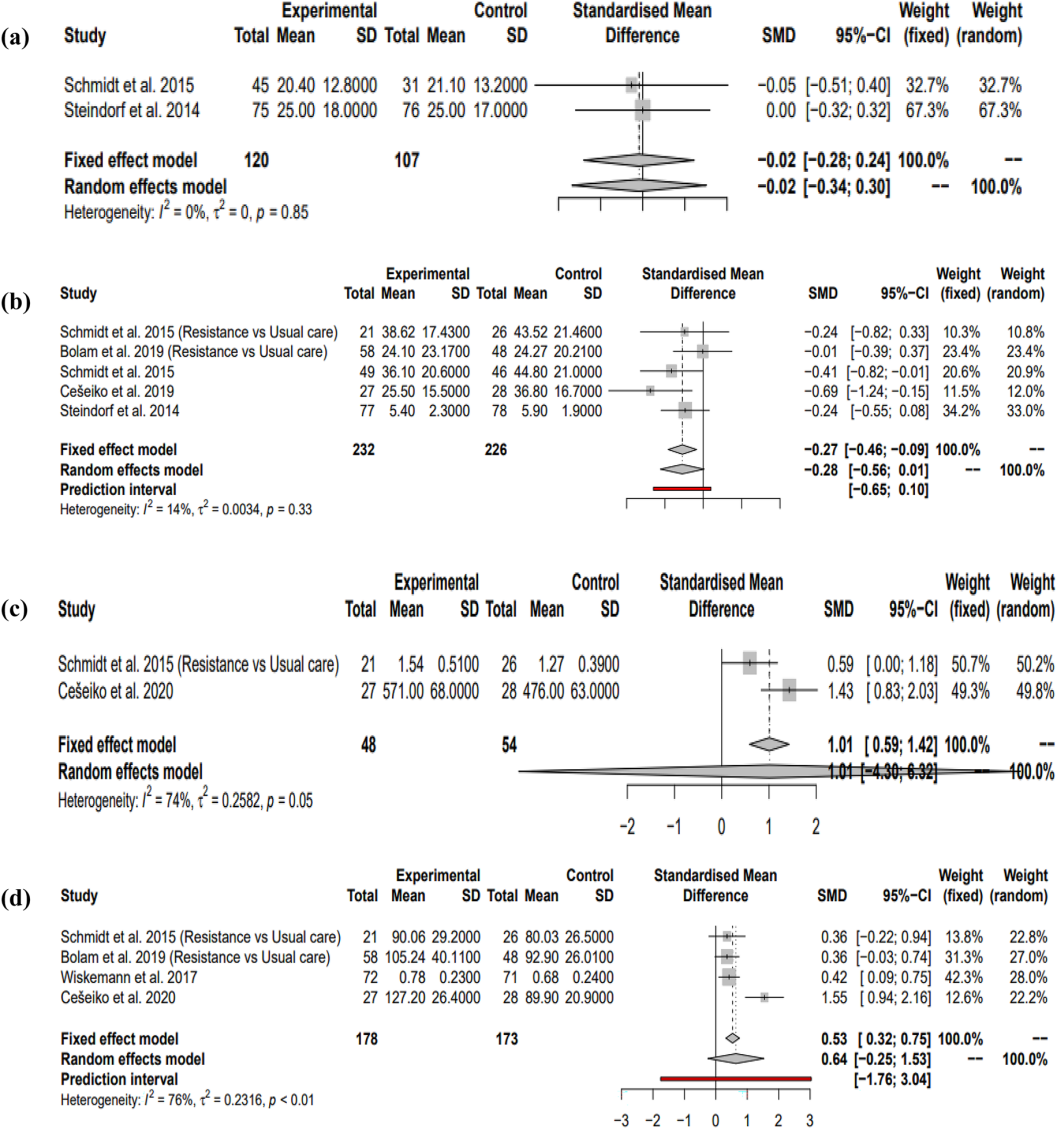

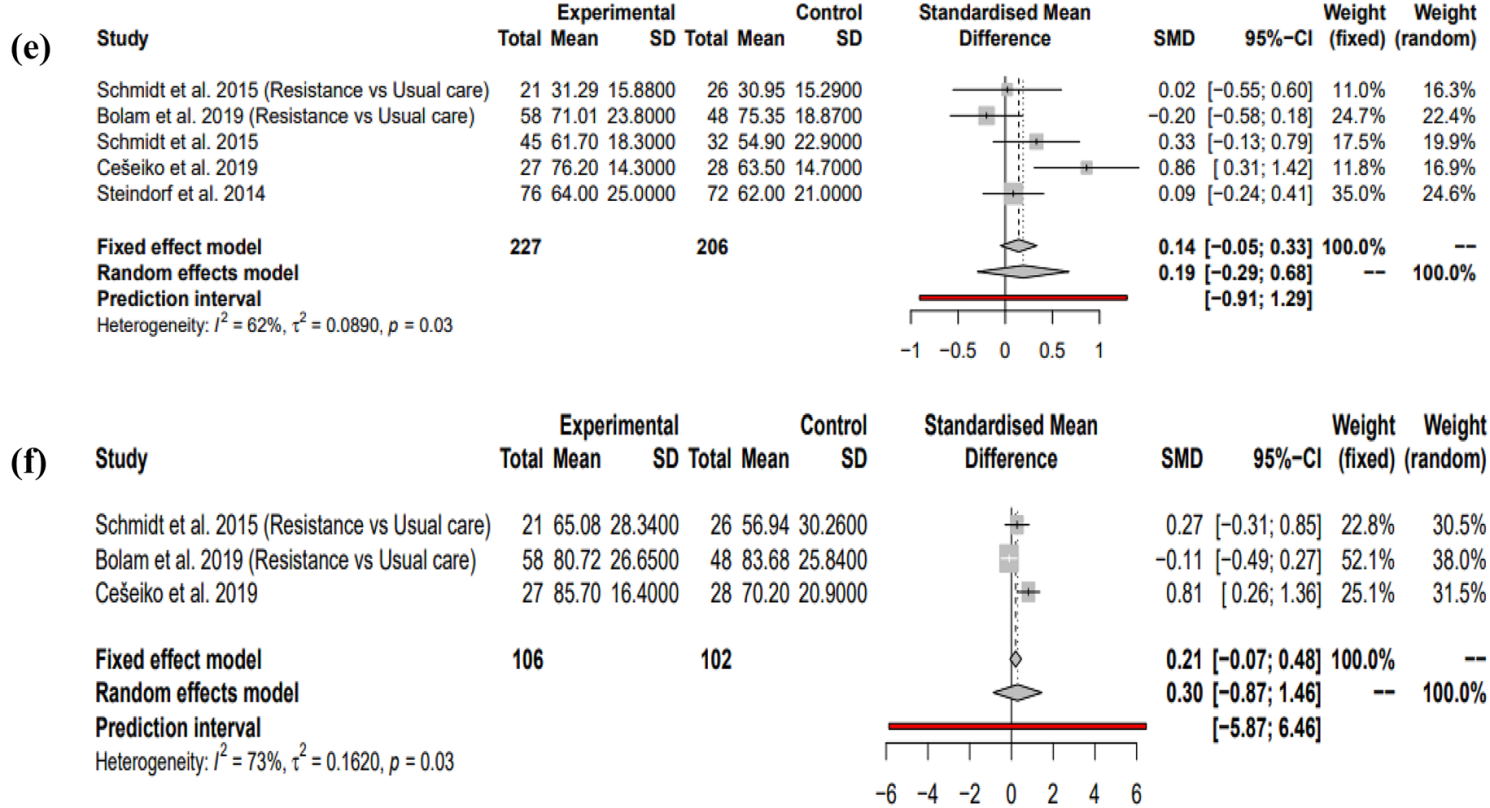
Random effects models showing the effects of resistance interventions on (**b**) global fatigue, (**c**) muscular endurance, (**d**) muscular strength, (**e**) QOL, (**f**) social functioning during adjuvant treatment. Fixed effects model showing the effects of resistance interventions on (**a**) depression during adjuvant treatment. Negative effect sizes favour the exercise intervention in (**a,b**). Positive effect sizes favour the exercise intervention in (**c**,**d**,**e,f**).

Two studies^22,23^ were found that matched the inclusion criteria were used to investigate the long-lasting effects of resistance exercise on depression in female breast cancer patients undergoing adjuvant therapy. Reporting the fixed effects model, collectively they showed a non-significant small negative effect size (SMD = − 0.02, 95% CI = [− 0.28; 0.24], I^2^ = 0%, *P* = 0.895). This meta-analysis however had low power at 5.26%. This is shown in Fig. 6a.

#### Interventions comprising solely resistance exercise: global fatigue

Five studies^20,22,23,27,28^ were found to be eligible using the inclusion criteria to investigate the effects of resistance interventions on global fatigue. All studies displayed negative effect sizes giving a non-significant negative overall effect size using the random effects model (SMD = − 0.28, 95% CI = [− 0.56; 0.01], I^2^ = 14%, *P* = 0.055). Prediction intervals also favour this. Power analysis showed sub-optimal power to detect significance where it exists using these studies at 74.05% (Fig. 6b).

#### Interventions comprising solely resistance exercise: muscular endurance

Two studies^28,29^ were suitable to quantify the enduring effects of resistance interventions on muscular endurance during adjuvant therapy for breast cancer. Collectively, reporting the random effects model, a large non-significant positive effect size was observed, favouring the intervention, with high power at 96% (SMD = 1.01, 95% CI = [− 4.30; 6.32], I^2^ = 74%, *P* = 0.25). However, due to the lack of studies to investigate this relationship, prediction intervals could not be performed. This is demonstrated in Fig. 6c.

#### Interventions comprising solely resistance exercise: muscular strength

Four studies^20,28–30^ that matched the inclusion criteria were used to quantify the effects of resistance interventions on muscular strength during adjuvant treatment. All four studies showed positive effect sizes favouring the intervention and gave a cumulative moderate positive effect size using the random effects model (SMD = 0.64, 95% CI = [− 0.25; 1.53], I^2^ = 76%, *P* = 0.11). Prediction intervals suggest future studies will also obtain similar findings and power analysis shows optimal power to detect a significant effect size where one exists at 98.62%. This was however not statistically significant (Fig. 6d).

#### Interventions comprising solely resistance exercise: QOL

Five studies were found to be eligible for this meta-analysis^20,22,23,27,28^. Four studies exhibited positive effect sizes with the other was negative. Together reporting the random effects model, they gave a low positive non-significant effect size (SMD = 0.19, 95% CI = [− 0.29; 0.68], I^2^ = 62%, *P* = 0.33). Prediction intervals also reflect this. Power analysis showed there to be poor power to detect a significant effect size at 33.64% (Fig. 6e).

#### Interventions comprising solely resistance exercise: social functioning

Using the random effects model, cumulatively, three studies^20,27,28^ showed a low positive effect size when investigating the effects of resistance interventions on social functioning (SMD = 0.30 95% CI = [− 0.87; 1.46], I^2^ = 73%, *P* = 0.39). Power analysis showed poor power (38.68%), with prediction intervals being very broad so displayed no clear direction. This is shown in Fig. 6f.

#### Interventions comprising solely endurance exercise: cardiorespiratory fitness

**Figure 7:**
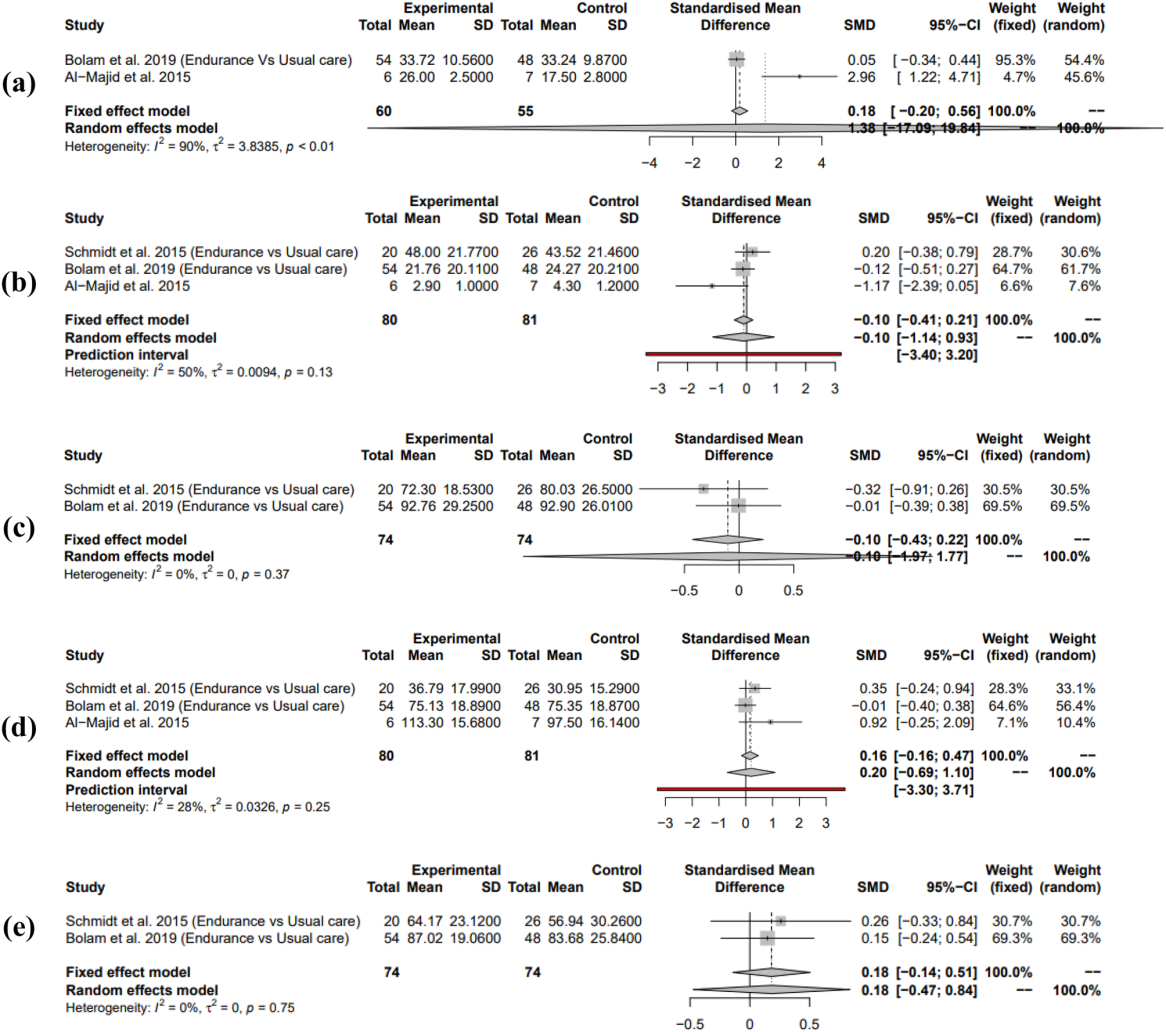
Random effects models showing the impact of endurance interventions on (**a**) cardiorespiratory fitness, (**b**) global fatigue, (**d**) QOL during adjuvant treatment. Fixed effects models showing the impact of endurance interventions on (**c**) muscular strength, (**e**) social functioning. Positive effect sizes favour the exercise intervention in (**a**,**c**,**d,e**). Negative effect sizes favour the exercise intervention in (**b**).

Two studies^19,20^ were used to quantify the effects of endurance interventions on cardiorespiratory fitness. Both of these showed positive effect sizes and together gave a large positive effect size when reporting the random effects model with optimal statistical power at 99.71% (SMD = 1.38, 95% CI = [− 17.09; 19.84], I^2^ = 90%, *P* = 0.52). Since only two studies were used, the 95% CI was very large and prediction intervals were not able to be synthesised (Fig. 7a).

#### Interventions comprising solely endurance exercise: depression

No studies were found to be eligible to investigate the effects of endurance interventions on depression.

#### Interventions comprising solely endurance exercise: global fatigue

Three studies^19,20,28^ were used to quantify the impact of endurance interventions on global fatigue during adjuvant therapy and collectively using the random effects model, they showed a non-significant low negative effect size (SMD = − 0.10, 95% CI = [− 1.14; 0.93], I^2^ = 50%, *P* = 0.71). This finding was however non-significant with low statistical power (7.82%). Prediction intervals show no definitive future direction (Fig. 7b).

#### Interventions comprising solely endurance exercise: muscular endurance

Only one study was available to be used to investigate the effects of endurance interventions of muscular endurance. Schmidt et al*.*^28^ gave a non-significant positive effect size (SMD = 0.37, 95% CI = [− 0.22; 0.96], *P* = 0.22). Prediction intervals and power analysis could not be carried out.

#### Interventions comprising solely endurance exercise: muscular strength

Two studies^20,28^ were used for this meta-analysis, both displaying negative effect sizes. Using the fixed effects model, the overall effect size was non-significant negative (SMD = − 0.10, 95% CI = [− 0.43; 0.22], I^2^ = 0%, *P* = 0.22). There was however low statistical power (9.33%) and no prediction intervals could be synthesised. This is shown in Fig. 7c.

#### Interventions comprising solely endurance exercise: QOL

Collectively, the three studies^19,20,28^ selected to investigate the effects of endurance interventions on QOL during adjuvant treatment showed a non-significant positive effect size when reporting the random effects model (SMD = 0.20, 95% CI = [− 0.69; 1.10], I^2^ = 28%, *P* = 0.43). There was however poor statistical power in this meta-analysis (19.63%). Prediction intervals showed no clear direction (Fig. 7d).

#### Interventions comprising solely endurance exercise: social functioning

Two studies were used to investigate the impact of endurance interventions on social functioning^20,28^. Both of these showed positive effect sizes and together gave a non-significant positive effect size when reporting the fixed effects model (SMD = 0.18, 95% CI = [− 0.14; 0.51], I^2^ = 0%, *P* = 0.27). This finding was non-significant with low statistical power (19.4%). Prediction intervals could not be generated (Fig. 7e).

### Resistance and endurance interventions vs endurance interventions alone

**Figure 8:**
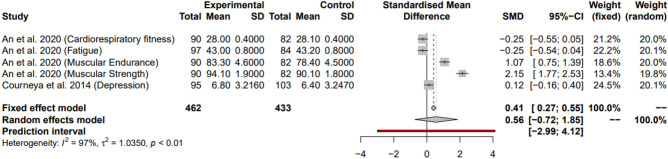
Random effects model showing the effects of adding resistance interventions to endurance interventions on adjuvant therapy related side effects compared to using endurance interventions alone. Positive effect sizes favour the exercise intervention.

To further explore which of the two interventions were better alone, two studies^21,24^ were used which both contained a ‘COMB’ (both resistance and endurance interventions) and a ‘STAN’ (endurance only) condition. The COMB was used as the exercise condition while STAN was used as the control. From the 7 outcome measures, 5 were available to measure. Overall using the random effects model, the meta-analysis gave a non-significant moderate positive effect size with optimal power (99.9%; SMD = 0.56, 95% CI = [− 0.72; 1.85], I^2^ = 97%, *P* = 0.29). Prediction intervals confirmed this for future studies (Fig. 8).

## Discussion

To our knowledge, this systematic review and meta-analysis is the first to date characterising the lasting effects of combined exercise interventions on physical fitness and mental wellbeing during adjuvant therapy using the factors investigated herein.

These meta-analyses show interventions consisting of both resistance and endurance exercise elicit significant long-lasting improvements in global fatigue (SMD = − 0.26, 95% CI = [− 0.46; − 0.07], I^2^ = 0%, *P* = 0.008). This is supported by Carayol et al*.*^38^ who also finds exercise interventions consisting of resistance, aerobic, and yoga exercise significantly improves fatigue in breast cancer patients receiving adjuvant therapy (*P* < 0.0001), which is important because high levels of both global and cancer-related fatigue during adjuvant treatment have been significantly linked to decreased adherence to treatment. Kidwell et al*.*^39^ showed this as patients who were feeling tired/fatigued had significantly decreased adherence to aromatase inhibitor adjuvant therapy compared to patients without this symptom (OR = 1.76). In addition, Ruddy et al*.*^40^ also demonstrated a link between cyclophosphamide-methotrexate-5-fluorouracil (CMF) treatment attrition rates and patient fatigue (*P* = 0.025). Therefore, this finding is of clinical value to reducing fatigue, enhancing treatment adherence and therefore efficacy, and improving disease prognosis.

A lack of studies investigating the effects of combined exercise interventions on muscular endurance and social functioning meant complete statistical analysis could not be completed, therefore warranting further investigation in future randomised controlled trials.

The four remaining factors (cardiorespiratory fitness, depression, muscular strength, and QOL) showed non-significant lasting improvements following interventions consisting of both resistance and endurance exercise. As such, overall, there are no statistically significant lasting effects of combined resistance and endurance interventions on physical fitness and mental wellbeing in female breast cancer patients (≥ 18 years old) undergoing adjuvant therapy compared to adjuvant therapy alone, which is summarised in Table 6 in the Supplementary Materials.

Despite being non-significant, these findings indicate there are still clinical benefits of combined exercise interventions to these adjuvant therapy side effects. Firstly, these findings show combined interventions elicit small improvements in cardiorespiratory fitness which is supported by other meta-analyses such as Furmaniak, Menig and Markes^41^ and Lahart et al*.*^42^ who show exercise interventions during and after adjuvant therapy non-significantly and significantly improve cardiorespiratory fitness respectively. This is reinforced by Wiestad et al*.*^43^ and Møller et al*.*^44^ who found exercise interventions elicit significant long-lasting improvements in cardiorespiratory fitness following adjuvant therapy. The present finding therefore implies combined exercise interventions enhance cardiorespiratory fitness which may contribute to enduring amelioration of physical fitness following adjuvant therapy. This is however modulated by ethnicity as shown by Dieli-Conwright et al*.*^45^ who found that patients of Hispanic origin had lower baseline cardiorespiratory fitness following adjuvant treatment. On balance, emerging evidence suggests exercise interventions should be tailored accordingly during adjuvant therapy to maximise the lasting clinical benefits to cardiorespiratory fitness and therefore physical fitness.

Secondly, the present findings indicate clinical benefits of combined interventions to muscular strength (shown by the 0.47 effect size), albeit g non-significant. Support for this is provided by two recent meta-analyses conducted by Lahart et al*.*^42^ and Møller et al*.*^44^ who both found combined exercise interventions elicit significant enduring improvements in muscular strength following adjuvant therapy. Thus, combined exercise interventions may offer long-lasting clinical amelioration of muscular strength when completed during adjuvant treatment contributing to enhanced physical functioning and clinical outcome.

The present study also demonstrates enduring clinical benefits of combined interventions to reducing depression, with an effect size of − 0.42. Meta-analyses by Carayol et al*.*^38^; Furmaniak, Menig and Markes^41^ and Lahart et al*.*^42^ support this by demonstrating significant enduring improvements in depression in response to combined exercise interventions during adjuvant treatment. These effects may also apply to exercise interventions completed following adjuvant therapy^46–48^.

Enhanced cardiorespiratory fitness and muscular strength, along with reduced global fatigue and depression may collectively contribute to enhanced physical fitness and mental wellbeing and therefore improved QOL as demonstrated by these meta-analyses, and supported by other studies^38,41,42,46,49,50^, who all found significant improvements in QOL following exercise interventions. Taken together, these findings clearly demonstrate the lasting benefits of combined exercise interventions on reducing the negative side effects from adjuvant therapy on physical fitness and mental wellbeing.

There are multiple mechanisms which may underly the depression which leads to decreased QOL and mental wellbeing arising from adjuvant therapy such as chemotherapy. Of importance, one such mechanism could be through a disruption in monoamine homeostasis^51^.This may be due to the non-specific nature of chemotherapy, causing damage associated molecular patterns to arise from both tumourigenic and healthy cells, which subsequently bind to pattern recognition receptors such as Toll-like receptors (TLRs) to stimulate pro-inflammatory pathways, including *NF*-*κB*^51^.As a result, pro-inflammatory cytokines such as TNF-α may increase the reuptake of several neurotransmitters including serotonin, dopamine, noradrenaline and bone-derived neurotrophic factor (BDNF) resulting in lower serum levels leading to symptoms of depression. Therefore, a mechanistic basis for these findings in improving mental wellbeing after exercise may lie in biochemical alterations to these monoamines in response to exercise. Research by Helmich et al*.*^52^ and Basso and Suzuki^53^ show exercise induces serum increases in serotonin, dopamine, norepinephrine and BDNF^54^. Therefore, it may be postulated that serum increases in monoamine levels following exercise interventions during chemotherapy may work to restore monoamine homeostasis alleviating depressive symptoms thus improving QOL.

A potential mechanism underlying the improvements in muscular strength, and therefore physical fitness, observed with combined exercise interventions may lie in leukocyte alterations following exercise. Generally, the role of leukocytes in muscle repair and hypertrophy is well characterised: in response to acute myotrauma, a pro-inflammatory response occurs, establishing a chemotactic gradient for leukocyte invasion. These leukocytes augment inflammation by secreting growth factors and cytokines to stimulate satellite cell recruitment for repair^55^. Alongside satellite cells, M2 macrophages assist in repair and hypertrophy by modulating inflammation and aiding in the formation of novel myofibers and myonuclei^56–58^. In healthy individuals, leukocyte levels are within the normal range meaning muscle regeneration after exercise occurs normally, however chemotherapy regimens in breast cancer patients can significantly decrease blood leukocyte counts^55^. This may result in impaired muscle repair following exercise, leading to decreased muscular strength and hypertrophy after completing daily tasks during adjuvant treatment. Over time, since repair is impaired, muscular strength and health may decline leading to decreased physical fitness during adjuvant treatment. This would not only account for why chemotherapy has detrimental effects on physical fitness but also why exercise interventions may improve muscular strength following adjuvant treatment. Furthermore, following exercise bouts, leukocyte counts significantly increase^59^ which may improve muscular regeneration and hypertrophy after exercise. Recent research also demonstrates epigenetic alterations in leukocytes favouring the demethylation and activation of anabolic pathways such as growth hormone-releasing hormone following exercise, thereby improving muscular hypertrophy and regeneration^60^. Thus, the beneficial effects of exercise interventions on muscular strength may be mediated by increased leukocyte counts and alterations in the leukocyte epigenetic landscape favouring hypertrophy and repair. To complement this, exercise interventions such as endurance exercise are well characterised to improve oxygen uptake, enhancing cardiorespiratory fitness, which may in turn result in higher muscle oxygenation and therefore enhanced performance, leading to enhanced physical strength and fitness following adjuvant therapy. Holistically, improving muscular strength and health is of clinical importance to avoid the development of sarcopenia which may be exacerbated by adjuvant therapies, thereby preventing the deterioration of physical fitness, QOL and mental wellbeing^61,62^.

The present findings also show interventions consisting of solely resistance exercise have an enduring, albeit non-significant, effect on improving each of the factors, apart from depression where there is little/no effect, which aligns with previous meta-analyses^63,64^ (Table 3). The present study also suggests endurance interventions improve each factor, except muscular strength in which it has a small negative impact. One explanation for this unexpected result could be that endurance interventions elicit high levels of autophagy resulting in muscle protein breakdown exceeding synthesis leading to loss of muscle mass and strength^65^. However, the current paradigm based on an array of research suggests the opposite in that autophagy is key for muscle maintenance and homeostasis. Therefore, an alternative mechanism may be that endurance interventions induce transient muscle fibre type transitions from type II to type I fibres over the intervention period, increasing muscular endurance at the expense of muscular strength^66^. Taken together, these findings indicate that resistance exercise interventions are more effective than endurance exercise to lastingly improve adverse side effects from adjuvant therapy when performed alone. This is evident in both the separate and comparative (fourth group) meta-analyses.

**Table 3:** Other meta-analyses that support present findings.

Study	Findings used in our study
Carayol et al.^38^	Exercise significantly improved fatigue, depression, and QOL This effect was highest in lower doses
Furmaniak, Menig and Markes^41^	Although non-significant exercise can slightly improve cardiorespiratory fitness, depression and QOL
Lahart et al.^42^	Exercise significantly enhanced cardiorespiratory fitness, lower body muscular strength, depression scores, and QOL
Patsou et al.^47^	Overall exercise can non-significantly improve depressive symptoms When broken down into aerobic and resistance exercise, aerobic exercise had significant effects while resistance did not
Lee and Lee^49^	Exercise elicited small improvements in QOL
Cheema et al.^63^	Exercise significantly enhanced lower body muscular strength After removing two studies, exercise also significantly increased QOL
Padilha et al.^64^	Resistance exercise significantly improved lower body muscular strength

### Limitations

Despite deploying methodology to minimise bias, there are still some important limitations to consider. Firstly, some of these meta-analyses are negatively impacted by studies with small sample sizes. Alongside this, multiple analyses suffer from high heterogeneity which together, may lead to low statistical power. This may lead to type 2 errors and bias resulting in the possibility of misinformed conclusions. In addition, some of these meta-analyses are limited by study availability due to authors not replying with the required information and due to a lack of research in these areas. The possibility of missed papers during study selection also cannot be ruled out, although rigorous measures were taken to minimise this risk. In addition, the future direction provided by some prediction intervals were not clear, possibly impeding conclusions. These limitations therefore warrant further research into some of these adjuvant therapy factors to further inform clinical recommendations during adjuvant therapy.

### Future research

These findings indicate that due to a lack of studies, more research is required in the following areas: the effects of combined interventions on depression, muscular endurance and social functioning, the effects of resistance interventions on cardiorespiratory fitness, depression and muscular endurance, and the effects of endurance exercise on cardiorespiratory fitness, depression, muscular endurance, muscular strength, and social functioning. Additionally, due to a lack of power and non-definitive prediction intervals, further research is warranted in the following areas: the effects of combined interventions on QOL, the effects of resistance interventions on QOL and social functioning and finally, the effects of endurance interventions on global fatigue and QOL.

In conclusion, the findings presented within show combined exercise interventions elicit significant enduring benefits to global fatigue during adjuvant therapy in breast cancer patients. They also suggest a lasting clinical benefit for combined interventions to improving the remaining factors (cardiorespiratory fitness, depression, muscular endurance, muscular strength, QOL, and social functioning) thus improving physical fitness and mental wellbeing. When performed separately, these results suggest both types of interventions are beneficial in improving physical fitness and mental wellbeing. Finally, in the event combined interventions cannot take place, interventions consisting of solely resistance exercise elicit higher clinical benefits than endurance interventions alone.

## Supplementary Information

Supplementary Information.

## Data Availability

All data available from the published papers and the authors therein.

## Code availability

All base code can be found from the online version of “Doing Meta-Analysis With R: A Hands-On Guide”. Please refer to Reference^33^. Examples of modified versions of the code for these analyses are available upon request.
