# Supplementary material for: The lasting effects of resistance and endurance exercise interventions on breast cancer patient mental wellbeing and physical fitness
Source: Sci Rep. 2022 Mar 3;12:3504. doi: 10.1038/s41598-022-07446-3 (PMC8894392; doi:10.1038/s41598-022-07446-3)
Supplement: Supplementary file 1 — Supplementary Information. [file 41598_2022_7446_MOESM1_ESM.docx]

**Appendix**

**Table 4:** Primary search terms to obtain papers for this meta-analysis. Additional specific search terms used to minimize the chance that relevant papers were missed.

| Primary search terms | Additional specific search terms |
| --- | --- |
| “exercise” AND “cancer” | “resistance exercise” AND “breast cancer” AND “fatigue” |
| “endurance” AND “cancer” | “resistance exercise” AND “breast cancer” AND “social functioning” |
| “endurance” AND “cancer” AND “chemotherapy” | “resistance exercise” AND “breast cancer” AND “depression” |
| (“endurance” AND “resistance”) AND “cancer” | “resistance exercise” AND “breast cancer” AND “muscular strength” |
| (“endurance” AND “resistance”) AND “cancer” AND “chemotherapy” | “resistance exercise” AND “breast cancer” AND “muscular endurance” |
| (“endurance” AND “resistance”) AND “cancer” AND “chemotherapy” AND “quality of life” | “resistance exercise” AND “breast cancer” AND “cardiorespiratory fitness” |
| (“endurance” AND “resistance”) AND “cancer” AND “chemotherapy” AND “quality of life” AND “pathologies” | “endurance exercise” AND “breast cancer” AND “fatigue” |
| (“endurance AND resistance”) AND “cancer” AND “chemotherapy” AND “quality of life” AND “pathologies” AND “breast cancer” | “endurance exercise” AND “breast cancer” AND “social functioning” |
| (“endurance”) AND “cancer” AND “chemotherapy” AND “quality of life” AND “pathologies” AND “breast cancer” | “endurance exercise” AND “breast cancer” AND “depression” |
| “endurance” AND “resistance exercise” AND “breast cancer” | “endurance exercise” AND “breast cancer” AND “muscular strength” |
| (“endurance” AND “resistance exercise”) AND “cancer” AND “chemotherapy” AND “quality of life” AND “pathologies” AND “breast cancer” | “endurance exercise” AND “breast cancer” AND “muscular endurance” |

|  | Cardiorespiratory fitness | Depression | Global fatigue | Muscular endurance | Muscular strength | Quality of life | Social functioning |
| --- | --- | --- | --- | --- | --- | --- | --- |
| Group 1  Papers with interventions comprising both resistance and endurance exercise | Cornette et al. 2016 [20]; Dong et al. 2020 [21]; Cornette et al. 2013 [22]; Travier et al. 2015 [23]; Waart et al. 2015 [24]; Casla et al. 2015 [25] | Cornette et al. 2016 [20]; Travier et al. 2015 [23] | Cornette et al. 2016 [20]; Schmidt et al. 2012 [27]; Travier et al. 2015 [23]; Waart et al. 2015 [24]; Husebø et al. 2014 [26] | N/A | Cornette et al. 2016 [20]; Dong et al. 2020 [21]; Travier et al. 2015 [23]; Waart et al. 2015 [24]; Casla et al. 2015 [25] | Cornette et al. 2016 [20]; Schmidt et al. 2012 [27]; Travier et al. 2015 [23]; Casla et al. 2015 [25] | Dong et al. 2020 [21] |
| Group 2  Papers with resistance exercise interventions only | Bolam et al. 2019 [28] | Schmidt et al. 2015 [29]; Steindorf et al. 2014 [30] | Schmidt et al. 2015 [29]; Bolam et al. 2019 [28]; Schmidt et al. 2015 [31]; Cešeiko et al. 2019 [32]; Steindorf et al. 2014 [30] | Schmidt et al. 2015 [29]; Cešeiko et al. 2020 [33] | Schmidt et al. 2015 [29]; Bolam et al. 2019 [28]; Wiskemann et al. 2017 [34]; Cešeiko et al. 2020 [33] | Schmidt et al. 2015 [29]; Bolam et al. 2019 [28]; Schmidt et al. 2015 [31]; Cešeiko et al. 2019 [32]; Steindorf et al. 2014 [30] | Schmidt et al. 2015 [29]; Bolam et al. 2019 [28]; Cešeiko et al. 2019 [32] |
| Group 3  Papers with endurance exercise interventions only | Bolam et al. 2019 [28]; Al-Majid et al. 2015 [35] | N/A | Schmidt et al. 2015 [31]; Bolam et al. 2019 [28]; Al-Majid et al. 2015 [35] | Schmidt et al. 2015 [31] | Schmidt et al. 2015 [31]; Bolam et al. 2019 [28] | Schmidt et al. 2015 [31]; Bolam et al. 2019 [28]; Al-Majid et al. 2015 [35] | Schmidt et al. 2015 [31]; Bolam et al. 2019 [28] |
| Group 4  Papers with interventions comprising both resistance and endurance exercise or with endurance only as the control | An et al. 2020 [36] | Courneya et al. 2014 [37] | An et al. 2020 [36] | An et al. 2020 [36] | An et al. 2020 [36] | N/A | N/A |

**Table 5:** The 18 selected papers sorted into their respective meta-analysis groups

|  | Cardiorespiratory Fitness | Depression | Global Fatigue | Muscular Endurance | Muscular Strength | Quality of Life | Social Functioning |
| --- | --- | --- | --- | --- | --- | --- | --- |
| Both resistance and endurance interventions | x | x | ** | N/A | x | x | x |
| Solely resistance interventions | x | x | x | x | x | x | x |
| Solely endurance interventions | x | N/A | x | x | x | x | x |
| Resistance and endurance interventions vs endurance interventions | x | x | x | x | x | N/A | N/A |

**Table 6:** A summary table of significance showing overall, there are no long-lasting improvements in physical fitness and mental wellbeing following resistance and endurance exercise interventions.

^x = non-significant^

- ^= p^ $\leq$ ^0.05^
- ^** p^ $\leq$^0.01^
